# Intimate partner violence after childbirth: an explanatory sequential mixed-methods study protocol

**DOI:** 10.1186/s12978-024-01825-x

**Published:** 2024-06-11

**Authors:** Fatemeh Ghelichkhani, Zahra Behboodi Moghadam, Armin Zareiyan, Masoumeh Namazi

**Affiliations:** 1 Department of Midwifery and Reproductive Health, School of Nursing and Midwifery, Tehran University of Medical Sciences, Tehran, Iran; 2 Department of Public Health, School of Nursing, Aja University of Medical Sciences, Tehran, Iran

**Keywords:** Intimate partner violence, Postpartum, Related factors, Mixed method, Qualitative content analysis, Childbirth

## Abstract

**Background:**

Intimate partner violence (IPV) is the most common form of violence against women. Postpartum IPV refers to any type of IPV that occurs up to one year after childbirth and has many adverse impacts on mothers and their children. Considering the lack of sufficient information on the prevalence and factors related to IPV after childbirth in Iran, this study aimed to evaluate the frequency and severity of IPV, its different forms, and psychosocial factors related to IPV, as well as to explore how IPV is perceived among mothers one year after childbirth.

**Methods:**

An explanatory sequential mixed-methods design was used to conduct this study in two phases. The first phase is a cross-sectional study that will be performed on postpartum mothers who have a one-year-old child referred to health care centers in the southern region of Tehran, Iran, with the aim of determining the prevalence of IPV and its related factors. The second phase is a qualitative conventional content analysis study with the purpose of exploring women’s experiences and perceptions of IPV and its preventive or protective factors. Purposive sampling will be used. Based on the results of the quantitative phase, mothers who are at the two ends of the IPV spectrum (based on their total Conflict Tactics Scale (CTS-2) scores) will be selected, and in-depth and semistructured interviews will be conducted with them. Finally, the researchers will provide an interpretation of the quantitative results using qualitative data.

**Discussion:**

This is the first study that uses a mixed methods approach to explain different dimensions of IPV, its related factors, and mothers' perceptions of it. By providing a better understanding of this phenomenon, it is hoped that the results of this research will be used by policymakers and officials of educational and cultural systems to plan and provide effective interventions, enact laws, and present educational and cultural programs to prevent IPV after childbirth.

**Ethical code:**

IR.TUMS.FNM.REC1400.200.

## Abstract (plain language summary)

Intimate partner violence is the most common form of violence against women, with a higher prevalence in Asian and Eastern Mediterranean countries, including Iran. The period after childbirth is a stressful and anxiety-provoking event due to the increased physical, psychological, social, and economic needs of parents, leading to intimate partner violence. Studies show that women who experience violence after childbirth face many physical, sexual, and emotional problems. Additionally, it will have a negative impact on their baby. Many factors can lead to intimate partner violence, among which psychological factors are among the most important. Therefore, we decided to perform a study in two phases with the aim of determining the prevalence and factors related to intimate partner violence. In the first phase, we will study Iranian women who have a one-year child living in southern Tehran city and ask them to complete questionnaires related to intimate partner violence. In the second phase, we will conduct an interview with women who have experienced violence after childbirth to obtain more information about the factors involved.

## Background

Intimate partner violence (IPV) is the most common complicated form of violence against women [1], affecting 13–61% of women worldwide, with a higher prevalence in Asian and Eastern Mediterranean countries, including Iran [2].

Postpartum IPV refers to any type of IPV that occurs up to one year after childbirth [3]. The postpartum period and the transition to parenthood can be stressful and anxiety-provoking events due to the increased physical, psychological, social, and economic needs of parents [4, 5], leading to IPV [6]. In a study conducted in Iran, the overall prevalence of postpartum IPV was reported to be 58%, indicating a high prevalence of IPV after childbirth [7].

Many studies have emphasized that women who experience IPV after childbirth face many physical, sexual, and emotional problems [8–11]. In addition to mothers, IPV has negative impacts on infants, including sleep disorders [12, 13], breastfeeding deprivation [14, 15], being at risk for child abuse in the future [16], and social and emotional maldevelopment [17].

There are different reasons for increased IPV after childbirth, such as the challenge of caring for the newborn [18], unwanted pregnancy, disappointment of the husband with the gender of the baby [7], sleep deprivation of the mother, decreased sexual desire of the mother [19] and the inability of the mother to fully satisfy the husband’s sexual expectations [7, 20].

Exposure to IPV leads to the development or exacerbation of women's psychiatric disorders, such as depression, anxiety, and stress [21]. Among psychiatric disorders, depression is considered a predictor of IPV, and its screening helps to identify people at risk for IPV [22]. Most of the studies have considered depression as one of the consequences of IPV, but in a few studies, the impact of depression on the occurrence of IPV has been mentioned [22, 23]. Lipsky et al*.* showed that women's depression increases the probability of IPV by 3.25 times [22]. In another study conducted by Lee et al*.,* depression during pregnancy increased the probability of IPV by 2.17 times, and a history of psychological problems also increased the odds of IPV by 1.53 times [24]. Since IPV can lead to depression, and depression can be the basis for the formation of IPV, the relationship between them is complex [25]. Exposure to life stressors can also lead to IPV in women [26]. The period after childbirth can be particularly stressful for the mother due to the challenge of caring for the newborn and increases the risk of IPV, especially for mothers who have limited access to the necessary resources [18].

Resilience is a personality trait that helps a person adapt to environmental changes and stresses [27]. Jaffar Abbas et al*.* found that there is a significant relationship between resilience, stress, anxiety, and depression symptoms and IPV, and resilience can moderate it [28]. Studies on psychological factors related to violence are limited, and which psychological factors are most strongly related to the risk of IPV has not been discussed. Identifying the psychological factors associated with IPV and their impact on the occurrence of IPV will help to develop appropriate programs and interventions to prevent violence [29].

Most of the previous studies emphasized individual and/or interpersonal factors related to IPV. However, based on the socioecological model, IPV has a multidimensional identity [30, 31]. The social dimension, including the structure of society, rules, economic situation, people’s beliefs, and norms, is among the most important factors influencing IPV [32]. Social support is one of the factors that plays a very important role in reducing women's vulnerability to IPV [33]. Access to social support can moderate the consequences of IPV and improve mental health outcomes [34].

Insufficient awareness and lack of access to appropriate information on IPV after childbirth are major barriers to preventing IPV and improving maternal and child health [14]. The authors found only one study conducted in Iran that investigated the individual and interpersonal factors associated with IPV, specifically after childbirth [7]. Knowing the causal risk and protective factors of IPV is particularly important for designing interventions that can effectively reduce this problem [35]. Therefore, more studies with a more comprehensive perspective on this phenomenon need to be carried out to provide sufficient scientific evidence concerning IPV after childbirth. On the other hand, given the multidimensionality and complexity of the issue of violence, quantitative studies must be combined with qualitative studies to explain the issue and find effective solutions and interventions for reducing IPV after childbirth [36].

Considering the relatively high prevalence of IPV after childbirth in Iran [7, 37] and the lack of supportive laws or effective solutions for protecting women in this sensitive stage of their lives against IPV, further research is needed. At the time of the writing of this study protocol, no mixed-methods research on IPV after childbirth has been conducted in Iran to analyze and explain it. Therefore, we will conduct a mixed-methods study to evaluate the frequency and severity of IPV, its different forms, and psychosocial factors related to IPV, as well as to explore how IPV is perceived among mothers one year after childbirth. The findings of this research will help us take a step, albeit to a very small extent, in analyzing and explaining IPV to develop screening programs and effective strategies promoting mothers' and children's health as the most valuable members of society.

### Study aim

This study aimed to assess the prevalence of IPV and its associated factors one year after childbirth and to explain women’s experiences and perceptions of IPV after childbirth.

### Specific objectives

The specific objectives of the first phase include the following:

- ✓ determine the frequency and severity of IPV (physical, sexual, psychological, and injury-inflicting violence) after childbirth;
- ✓ Determining the relationships between demographic factors and IPV after childbirth
- ✓ The relationships between resilience, depression, stress, anxiety, and perceived social support and IPV after childbirth were determined.
- ✓ Determining the strategies that mothers use to prevent the occurrence of IPV.

The specific objectives of the second phase include the following:

- ✓ Women’s experiences and perceptions of IPV and preventive or protective factors should be explained.

### Methods/design

**Fig. 1:**
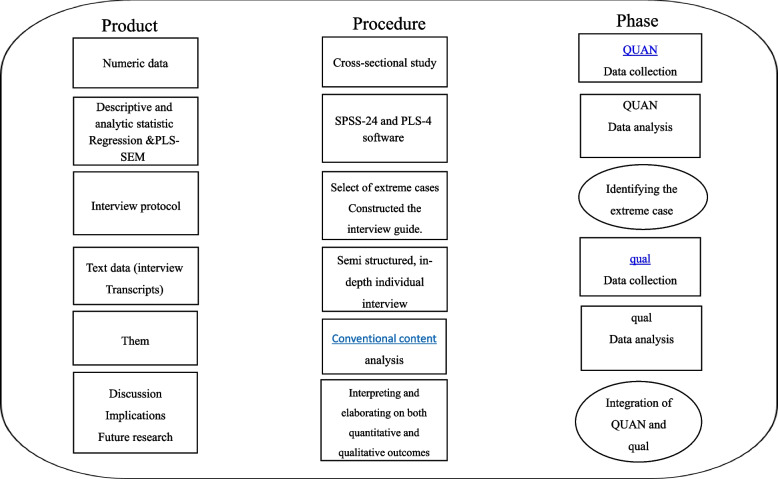
Diagram of the study process

The proposed research will be a mixed-methods sequential explanatory study. In this type of research, the data on the quantitative part of the study will be collected and analyzed first, and then, based on a need for further understanding of the quantitative results, the qualitative part will be used to explain and/or interpret the results of the quantitative phase. This type of study is very useful when the researcher intends to evaluate relationships between variables with quantitative data and to explain the reasons for these relationships [38]. The proposed study will be designed in two phases: the quantitative phase, followed by the qualitative phase. The study process diagram is depicted in Fig. 1.

### Phase 1: Quantitative study

#### Study design

In this phase, a cross-sectional study will be conducted on postpartum mothers who have a one-year-old child.

### Sampling

After obtaining approval from the Research Ethics Committee of Tehran University of Medical Sciences, 380 participants will be selected using cluster sampling from 87 healthcare centers in 5 districts in southern Tehran. First, healthcare centers in southern Tehran will be identified using the website of the South Tehran Health Center (sthn.tums.ac.ir), and two healthcare centers will be randomly selected from each district by lottery (a total of 10 healthcare centers). In the next step, the researchers will visit the selected healthcare centers to prepare a list of mothers who have one-year-old children in each healthcare center based on their electronic health records available on the SIB website (sib.tums.ac.ir). Then, mothers will be randomly selected from the list by the quota criterion using the www.random.org website, which generates random numbers between two numerical values entered on the site. The researchers will screen the selected mothers via telephone for inclusion and exclusion criteria, thoroughly explain the objectives of the research, and ask eligible mothers to participate in the study. Participants will be assured of the confidentiality of their personal information and the anonymity of the research reports. In the next step, the researchers will ask mothers to visit healthcare centers to complete self-report questionnaires. Considering the large number of questionnaire items, participants were informed that they could fill out the questionnaires at home and give them to the researchers on a specific date. In addition, the questionnaires will be coded to obtain the necessary information in the qualitative phase and to gain the participants’ trust. If they agree, a mobile number will be received from them, and a questionnaire link will be sent to them to complete at home.

### Inclusion criteria

- All Iranian women who live in southern Tehran, who have a one-year-old child, who have at least an elementary education, who have lived with their husband in the last year, and who do not have any mental disability or illness (mental disability, intellectual disability, or mental retardation) will be enrolled.

### Exclusion criteria

- Reluctance to continue participating in the research
- Failure to complete the questionnaires at any time during the research

### Scales and data collection

The quantitative data will be collected using the sociodemographic questionnaire, the Conflict Tactics Scale (CTS-2), the short form of the Connor-Davidson Resilience Scale (CD-RISC), the short form of the Depression, Anxiety, and Stress Scale (DASS-21), the Perceived Social Support Scale (PSSS), and two questions regarding the strategies mothers suggest to prevent and address domestic violence. A literature review suggested that the variables of resilience, social support, depression, stress, and anxiety may influence the occurrence or severity of violence against women [22, 23, 25–27, 34, 39, 40]. Therefore, the researchers used the above standardized and psychometrically approved questionnaires to measure these variables in the Iranian population.

- The sociodemographic questionnaire: This questionnaire included items such as age, age at marriage, duration of marriage, education level, employment status, forced marriage status, number of children, unwanted pregnancy status, spouse's age, spouse's education level, spouse's occupation, income sufficiency, infant's gender, satisfaction of the husband with the child's gender, and meeting the husband's sexual expectations.
- Conflict Tactics Scale (CTS-2): This standardized tool comprises 78 questions (39 paired items) that measure the prevalence and chronicity of IPV against men and women in the past year in five dimensions, including negotiation (6 items, emotional and cognitive subscales), psychological aggression (8 items), physical assault (12 items), sexual coercion (7 items), and injury (6 items). In this study, IPV against women was measured by employing 33 questions from the CTS2 in four dimensions (i.e., psychological aggression, physical assault, sexual coercion, and injury). Mild and severe forms of violence are also determined based on questions in each dimension. Eight response options (0–7) were available for measuring the frequency of IPV for each question. The scoring process is based on an average score for each response choice, as follows: 0 = never; 1 = once; 2 = twice; 3 = 3 to 5 times (the recommended midpoint is 4); 4 = 6 to 10 times (the recommended midpoint is 8); 5 = 11 to 20 times (the recommended midpoint is 15); 6 = more than 20 times (the recommended midpoint is 25); and 7 = not in the past 1 year but occurring before that. The presence of IPV is indicated by response options ranging from 1–6 for at least one question of any dimension, while the absence of 0 or 7 in all dimensions signifies that IPV is not present. By adding the average scores for minor and severe IPV questions on each dimension, the IPV chronicity score was calculated. The internal consistency of the CTS2 was high. According to reports, the Cronbach's alpha coefficients for different scales of its English version are between 0.79 and 0.95 [41]. Ardabily et al. translated the scales into Persian using the forward–backward method. The word “weapon” was excluded from the items (19, 20, 47, 48) because it is not compatible with Iranian culture. In addition, the test–retest method confirmed the reliability of this scale [42]. The validity and reliability of the CTS-2 have been confirmed in several studies [7, 42, 43].
- The short form of the Connor-Davidson Resilience Scale (CD-RISC): The original version of the CD-RISC was designed in 2003 to measure resilience, but Campbell-Sills and Stein (2007) developed a 10-item version of the scale in 2007. The items are scored on a five-point Likert scale from 0 to 4. The total score ranges from 0 to 40, with higher scores indicating greater resilience [44, 45]. The internal consistency of the 10-item version of the CD-RISC has been confirmed, with a Cronbach's alpha value of 0.85 [44]. In the study of Salimi et al*.* (2017), the content validity of the questionnaire was examined and adjusted by 12 experts and faculty members of the Faculty of Nursing and Midwifery at Tabriz University of Medical Sciences, and the results confirmed the content validity of the scale with a Cronbach's alpha value of 0.82 [46]. In addition, Ghorbani Sani et al*.* obtained a Cronbach's alpha coefficient of 0.98 for this instrument [47].
- Depression, Anxiety, and Stress Scale (DASS-21): The DASS-21 is an abbreviated form of the DASS-42, which was developed by Lovibond et al*.* to measure depression, anxiety, and stress. Stress, depression, and anxiety are the three subscales of the DASS-21, and items are scored on a four-point Likert scale from “not at all” (score 0) to “very high” (score 3). For each subscale, the minimum and maximum scores are 0 and 21, respectively, with higher scores indicating more severe depression, anxiety, and stress [48].
  Sahebi et al*.* confirmed the reliability of the DASS-21 for depression, anxiety, and stress subscales in the Iranian population, with Cronbach's alpha values of 0.81, 0.73, and 0.81, respectively. Moreover, the correlation coefficients between the depression subscale and the Beck Depression Inventory, the anxiety subscale and the Zung Self-Rating Anxiety Scale (SAS), and the stress subscale and the Perceived Stress Scale (PSS) were found to be 0.70, 0.67, and 0.49, respectively [49].
- Perceived Social Support Scale (PSSS): This multidimensional instrument will be used to examine social factors influencing IPV. The 12-item scale was designed by Zimet et al*.* (1988) to measure perceived social support provided by family, friends, and significant others. According to previous studies, the PSSS has good validity and desirable reliability [50]. In Iran, Salimi confirmed the overall reliability of the PSSS after obtaining Cronbach's alpha values of 0.86, 0.86, and 0.82 for the domains of family, friends, and a significant other, respectively [51].

### Sample size

Partial least squares structural equation modeling (PLS-SEM) will be used to investigate factors related to IPV. Therefore, according to the rule of thumb sample size calculation method, 2 to 5 participants must be enrolled for each manifest variable, and the minimum required sample size will be 250 to 300 individuals [52]. Since the researchers examined a total of 76 manifest variables (CTS-2: 33 items, CD-RISC: 10 items, DASS-21: 21 items, and PSSS: 12 items), the final sample size was 380 by considering 5 participants for each manifest variable.

### Analysis

**Fig. 2:**
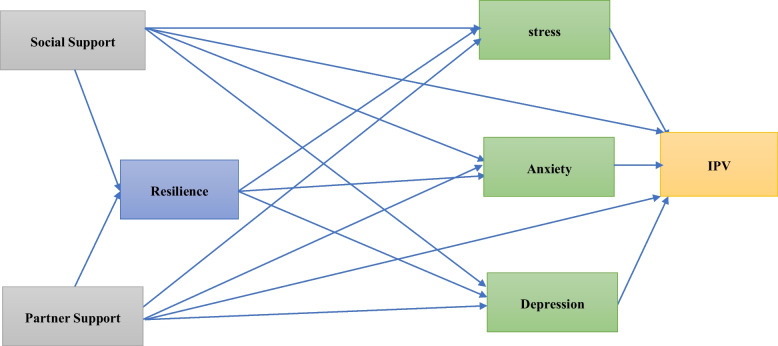
Path model of associations between psychosocial factors and IPV with the resilience mediator

The study will use IBM SPSS Statistics version 24 and PLS-4 software for data analysis. We will use descriptive statistics (frequency, percentage, mean, and standard deviation) to depict the demographic and psychosocial characteristics of the participants. The chi-square test, independent samples t test, and binary logistic regression were applied to investigate the correlations of demographic and psychosocial variables such as resilience, depression, stress, anxiety, perceived social support, and postpartum partner support with IPV. Bivariate binary logistic regression will be used to establish the unadjusted correlation between demographic and psychosocial characteristics and the prevalence of any form of IPV. Independent variables that have *P* values less than 0.2 in the bivariate tests will be incorporated into the multivariate binary logistic regression model with the Enter strategy to measure each of the independent variables' effects on the prevalence of IPV and to calculate the adjusted odds ratio (OR) and 95% CI. We are interested in assessing the impact of psychosocial factors (resilience, depression, stress, anxiety, perceived social support, and postpartum partner support) on IPV; thus, in the first step, based on a review of previous literature, a conceptual model will be drawn, and hypothesized associations will be tested with path models using PLS-SEM for model assessment. PLS-SEM is a new method for data analysis that has been widely accepted by researchers in all disciplines related to humanities studies, including medical sciences [53]. The proposed model for investigating the relationships between these psychosocial factors and IPV is shown in Fig. 2.

### Phase 2: Qualitative study

#### Study design

In the next phase, the researchers will conduct a study using conventional qualitative content analysis to thoroughly explain women's perceptions of IPV one year after childbirth. The results of the quantitative phase that need further explanation will be determined and used to design the research questions in the qualitative phase.

### Sampling

After providing informed consent from the participants, qualitative data will be collected via semistructured, in-depth individual interviews. Mothers who are willing to participate in the study will be selected through purposive sampling at the two ends of the IPV spectrum (based on their total CTS-2 scores). Before conducting the research, the researchers will prepare the interview guide and discuss how to focus on the research questions to obtain reliable data. They will also ask more in depth, exploratory questions based on participants' responses to obtain additional information. These are a few examples of interview questions:

- Please describe the current state of your life (in the period after childbirth).
- Has your husband displayed any particular behaviors or attitudes that hurt you in the postpartum period? Do you remember any issue that has upset or annoyed you during the year after childbirth?
- Explain how your husband treats you after childbirth.
- How do you explain your husband’s feelings (sad vs. happy) about birth? What was the sex preference of your partner? How does this affect your relationship with your husband?
- What are the risks and protective factors for IPV after childbirth?
- What psychosocial factors can lead to IPV after childbirth? Please explain about them.
- What solutions do you offer to reduce IPV after childbirth?

After establishing communication with the participants and providing a brief explanation concerning the importance of the research, the researchers conducted the interviews at a place and time convenient for the participants. The duration of the interviews will depend on how willing the participants are to continue the discussion and describe their experiences.

The interviews will be recorded with the permission of the participants and then transcribed. The participants will later be shown the transcribed interviews to control their accuracy. As the interviews continue, new questions may be added to the interview guide. In other words, the questions in semistructured interviews are not fixed in most cases but are flexible and shaped based on the course of the interview. The interviews will continue until the data are saturated (i.e., no newer information/codes can be derived from the interviews).

### Inclusion criteria

- Willingness to participate in the study
- Participants at the two ends of the IPV spectrum (based on their total CTS-2 scores)

### Exclusion criteria

- Mothers who experienced stressful events during the study (including divorce, death, or the diagnosis of an incurable disease of a close family member) or who were diagnosed with mental illness that prevented them from participating in an interview session(s) were excluded from the study.

### Data analysis

After completion and verbatim transcription of the interviews, the obtained data will be analyzed in MAXQDA 18 software using a conventional qualitative content analysis approach. The content analysis of the interviews will be performed according to the eight-step method proposed by Zhang and Wildemuth to analyze the content of the qualitative data and determine the themes and categories that explain the findings of the quantitative phase of the study [54]. This method includes the following steps:

1. Preparing data for qualitative analysis: Transcripts were read several times to gain a deep and comprehensive understanding of IPV after childbirth.
2. Defining data analysis units: Coding the text by specifying meaningful units (paragraphs, sentences, phrases, and words) and ensuring the preservation of meaning and content.
3. Classification: Placing codes in subcategories due to similarity and developing categories based on the relationships among subcategories.
4. Testing the coding pattern in a text sample: controlling the stability of the coding by two members of the research team and resolving disagreements in coding and categorization.
5. Coding the whole text of the interview: Expanding the code testing process to the entire text
6. Assessment of the coding stability: Rechecking the consistency of the coding after full-text encoding
7. Extracting conclusions from the coded data: finding the specifications and dimensions of the categories and identifying the relationships between them.
8. This paper presents a report of the findings [55].

Finally, the researchers will combine the findings of the quantitative and qualitative phases to present a comprehensive interpretation of the results, gain a better understanding of the phenomenon of IPV after childbirth and its associated factors, and suggest suitable strategies to prevent and reduce it.

### Ethics approval and consent to participate

Written informed consent will be obtained from all participants in both the quantitative and qualitative phases. In addition, participants will be assured of the confidentiality of their information and their right to withdraw from the study at any time. They will also be informed that their withdrawal from the study will not affect the quality of services that healthcare centers provide to them or their children. Participants who are diagnosed with psychological disorders during the research will be referred to counselors and psychologists.

## Discussion

IPV is a major social, legal, and health problem [56] that reflects gender inequality in societies [2]. The first year after childbirth is a sensitive and high-risk period for the occurrence of IPV or the intensification of previous abusive relationships, which have numerous negative impacts on the health of the mother and her child [57].

A few studies that have specifically investigated IPV after childbirth and its associated factors in Iran have revealed the high prevalence of this behavior among Iranian women. According to Motlagh et al*.*, postpartum IPV is more prevalent than reports suggest, but issues such as cultural limitations, poor communication, and the insensitivity of healthcare providers typically discourage victims from reporting cases of violence [58]. In a cross-sectional study, Amiri et al*.* reported that IPV is highly prevalent one year after childbirth. They also identified individual and interpersonal predictors of IPV [7]. The proposed study has several strengths and innovations. For example, a mixed-methods research design will be used to explain IPV, determine various types of IPV and their prevalence, and identify psychosocial factors associated with this phenomenon. In the quantitative phase of the study, the PLS-SEM method will be used to investigate psychosocial factors related to IPV. This method allows researchers to construct a network of complex relationships to examine the interrelationships among a group of variables [59]. To the best of our knowledge, our proposed study is the only one that examines the effects of common psychological factors in society, such as depression, anxiety, and stress, on the likelihood of IPV through path analysis. In addition, the qualitative phase of the study will provide researchers with invaluable information about women’s perceptions and experiences of IPV after childbirth. Additionally, this research will investigate IPV against women in the southern regions of Tehran. Considering the lower cost of living in southern regions than in other regions of Tehran, many people from different provinces and ethnic groups have migrated to these regions for marriage, education, or work. Therefore, it could be argued that the participants in the proposed research represent the entire population of Iranian women, and the results of this research can be generalized to all Iranian women. Nevertheless, there is a possibility of some biases related to the proposed study, such as recall bias when answering questions related to IPV or wish bias, where participants will try not to answer specific questions correctly. Considering the importance and value of the family in Iranian culture and the need to preserve the privacy of the family, we will probably face challenges in obtaining the consent of the mothers to participate in the research.

The proposed study will provide a comprehensive overview of different aspects of IPV after childbirth. Creating a better understanding of IPV after childbirth and the psychosocial factors associated with it, in this critical period of mothers' lives, will help the relevant authorities and policymakers implement effective measures, enact strict laws, and organize appropriate educational and cultural programs for all community members to reduce the occurrence of IPV, thereby improving the overall health and well-being of women and their children.

In addition, revealing and highlighting women's perceptions of IPV and its associated factors after childbirth can enhance multisectoral collaboration at all levels and provide a basis for designing interventions to reduce IPV.

## Data Availability

No datasets were generated or analysed during the current study.

## Abbreviations

CD-RISC: Connor-Davidson Resilience Scale
CTS-2: Conflict Tactics Scale
DASS: Depression, Anxiety and Stress Scale
IPV: Intimate Partner Violence Scale
PLS-SEM: Partial least squares structural equation modeling
PSSS: Perceived Social Support Scale
